# Agreement between Fenton and intergrowth curves in assessing birth weight of preterm infants: Bland-Altman analysis by degree of prematurity and birth weight for gestational age

**DOI:** 10.1016/j.jped.2026.101575

**Published:** 2026-06-26

**Authors:** Louise P.M. da Cunha, Ana Cecília T. Santiago, Crésio de A.D. Alves

**Affiliations:** Universidade Federal da Bahia, Faculdade de Medicina, Salvador, BA, Brazil

**Keywords:** Preterm infant, Birth weight, Gestational age, Small for gestational age, Anthropometry, Growth charts

## Abstract

**Objective:**

To assess the agreement between the Fenton and INTERGROWTH charts in classifying birth weight for gestational age in preterm infants, considering prematurity degree and small/adequate/large-for-gestational-age (SGA/AGA/LGA) status individually and simultaneously.

**Methods:**

Retrospective cohort study including 2529 preterm infants admitted between 2018 and 2021. Gestational age was estimated from obstetric data or by the Capurro/Ballard methods. Birth weight-for-gestational-age Z-scores were calculated according to both charts. Agreement was analyzed overall and by subgroups defined by prematurity degree and SGA/AGA/LGA status using the Bland-Altman method, complemented by boxplots.

**Results:**

Overall agreement was reasonable (mean bias: −0.08; limits of agreement: −0.67 to 0.50), with slight overestimation of Z-scores by INTERGROWTH. Relevant variability was observed across subgroups. Disagreement extended across all prematurity strata with distinct patterns: greater dispersion in extremely and very preterm infants and more consistent but systematically biased differences in moderate-to-late preterm infants (bias: -0.12). The largest discrepancies occurred among SGA, especially in extremely preterm infants, among whom 50% had Z-scores beyond the limits of agreement. In this subgroup, Fenton tended to assign higher Z-scores, especially between 26 and 32 weeks of gestation, attenuating birth weight deficit severity. Agreement was considerable among AGA and LGA infants, especially in more mature preterm groups.

**Conclusion:**

Although overall agreement between the charts was acceptable, clinically relevant differences were observed across all prematurity strata, particularly among more immature SGA infants. The charts are not interchangeable at the individual level, and their use may influence nutritional diagnosis and clinical management, reinforcing the importance of consistent and context-appropriate selection of growth reference.

## Introduction

The assessment of intrauterine growth is essential in neonatal care, guiding the diagnosis of intrauterine growth restriction (IUGR), nutritional planning, and risk stratification [1]. Birth weight for gestational age (GA) is the indicator that allows classification of newborns as small-for-gestational-age- (SGA), appropriate (AGA), or large-for-gestational-age (LGA), thereby guiding early interventions [2].

Several growth charts have been developed for nutritional assessment of preterm infants both at birth and thereafter, taking into account population, methodological, and epidemiological specificities. However, there is still no consensus regarding the choice of the most appropriate reference, especially for extremely preterm infants [3].

Charts such as those of Fenton (2013) and INTERGROWTH (2014) are widely used in clinical practice. The Fenton chart was based on a systematic review of large studies from developed countries using cross-sectional measurements of size at birth, whereas INTERGROWTH was constructed from an international cohort of low-risk pregnancies, with a small number of extremely preterm infants included [4,5]. Methodological differences between these charts affect the estimation of percentiles and Z-scores, potentially leading to practical impacts on the identification of SGA births and on nutritional planning [6,7].

Greater divergence between the charts has been reported in vulnerable subgroups, particularly in the presence of maternal risk factors (multiparity, chorioamnionitis, preeclampsia, gestational diabetes, and chronic diseases) [8]. Although numerous studies have compared chart agreement in determining outcomes (e.g. SGA classification) using concordance coefficients, to our knowledge, none have applied formal graphical agreement analysis, such as Bland-Altman or considered stratification by clinically relevant subgroups (degree of prematurity, SGA/AGA/LGA status) [[8], [9], [10], [11], [12], [13], [14]]. Notably, the clinical impact of variance between charts remains poorly characterized.

Thus, this study analyzed the agreement between the Fenton and INTERGROWTH charts in the assessment of birth weight for GA in a cohort of preterm infants, applying a formal graphical agreement methodology with analyses stratified by prematurity degree and SGA/AGA/LGA status (individually and simultaneously), to elucidate the implications of growth reference selection for nutritional assessment in this population.

## Methods

This was an observational study of diagnostic agreement based on a retrospective cohort of preterm infants, whose data were extracted from the national QualiNeo database, linked to the Brazilian Ministry of Health. Newborns admitted between January 2018 and December 2021 to three neonatal intensive care units (NICUs) and/or conventional intermediate care units (UCINCOs) of public hospitals in a Brazilian capital city were included.

Sex, birth weight, and gestational age at birth were recorded on standardized forms completed by the care team, as part of a larger project to monitor the quality of neonatal care.

Gestational age was estimated primarily from the date of the last menstrual period and first-trimester ultrasound; when unavailable, the Capurro or Ballard clinical methods were used. Birth weight was used to calculate the birth weight-for-gestational-age indicator, expressed as percentiles and Z-scores according to the Fenton (2013) and INTERGROWTH (2014) charts, using calculators available at: https://ucalgary.ca/resource/preterm-growth-chart/calculators-apps and http://intergrowth21.ndog.ox.ac.uk/.

Newborns were classified by birth weight adequacy as small for gestational age (SGA; < p10), appropriate for gestational age (AGA; p10–90), or large for gestational age (LGA; > p90) [15]. Prematurity was classified per World Health Organization criteria as extremely preterm (< 28 weeks), very preterm (28 to < 32 weeks), or moderate to late preterm infants (32–37 completed weeks) [16].

Term infants, preterm infants admitted exclusively to the Kangaroo Intermediate Care Unit, and those hospitalized for fewer than 14 days were excluded to select a clinically vulnerable population at higher nutritional risk, in whom differences in nutritional classification would be most relevant. Infants with congenital malformations or genetic syndromes were retained for the same reason. Those who died during hospitalization were excluded, as weight measurement at the study time point (required for future longitudinal analyses) would not be available.

**Figure 1:**
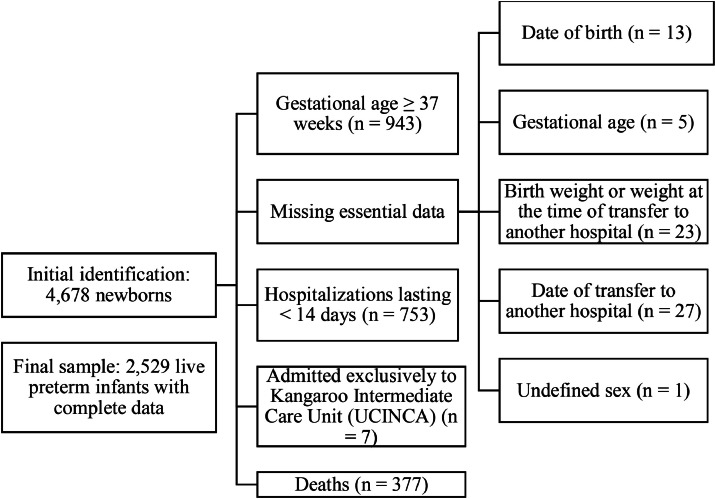
Study population definition.

The definition of the study population is shown in Figure 1.

Statistical analyses were performed using R software (version 4.5.0, R Core Team, Vienna, Austria). Sample characteristics were described using descriptive statistics - means and standard deviations (SD) for continuous variables and proportions for categorical variables.

Agreement between charts was assessed using Bland-Altman plots [17]. For each individual, the mean of the indicator values was plotted on the X-axis and the difference on the Y-axis (value of the Fenton indicator minus that of INTERGROWTH for each patient, in this order), such that more similar values result in points closer to zero. The mean difference (solid line), or mean bias, represents the systematic difference between methods (i.e., how much one method tends to measure more or less than the other on average; a value of zero indicates no systematic difference, whereas a non-zero value indicates consistent over- or underestimation). The limits of agreement (dotted lines) are defined as the mean difference ±1.96SD, representing the interval within which 95% of the differences between measurements are expected to lie under a normal distribution. The clinical validity of using both charts interchangeably depends, among other factors, on the mean bias, these limits, and whether they are considered clinically acceptable. Visual inspection allowed identification of dispersion patterns - asymmetric or distant from the mean line distributions, suggesting a lower agreement.

Analysis was performed for the whole group and stratified by clinical subgroups: degree of prematurity (extremely preterm, very preterm, and moderate-to-late preterm infants) and SGA/AGA/LGA status at birth, individually and simultaneously. The analysis considering only the SGA, AGA, and LGA subgroups was presented in a single plot; however, the values of mean bias, SD, and limits of agreement were calculated separately for each subgroup. Additionally, boxplots were constructed to assess the distribution of differences according to different gestational ages (in weeks).

The study was approved by the Research Ethics Committee of the participating institution (CAAE: 51,489,621.6.0000.5543) in accordance with current ethical principles for secondary data use, with consent requirement waived accordingly.

This research received no specific funding from public, commercial, or not-for-profit agencies.

## Results

The study population's general characteristics are summarized in Table 1.

**Table 1:** General characteristics of 2529 preterm infants assessed at birth between 2018 and 2021 are presented in mean (standard deviation) or frequency.

Variable		Mean	n	%	n	%
Gestational age at birth (weeks)		32 (2.6)				
Birth weight (grams)		1584.4 (489.5)				
Prematurity degree	Extremely preterm		195	7.7		
	Very preterm		813	32.1		
	Moderate to late preterm		1521	60.1		
Sex	Male		1352	53.5		
			Fenton		INTERGROWTH	
Birth weight-for-gestational-age classification		SGA	671	26.5	688	27.2
		AGA	1805	71.4	1767	69.9
		LGA	53	2.1	74	2.9

Agreement analyses between the methods are described below. Supplemental Figure 1 presents the overall Z-score agreement between the Fenton and INTERGROWTH charts, while Supplemental Table 1 summarizes the corresponding mean bias, standard deviation, and limits of agreement for all analyses. The mean bias was −0.08, near zero, indicating good overall agreement. The negative value reflects a slight overestimation of Z-scores by INTERGROWTH. The SD was 0.29, with limits of agreement of −0.67 to +0.50. Greater dispersion was observed at the lower end of the X-axis (Z-score < −2), while the range between −1.5 and +1.5 showed less variation. No cases fell below the lower limit of agreement, although a concentration was noted near and above the upper limit at the lower extreme of the X-axis.

**Figure 2:**
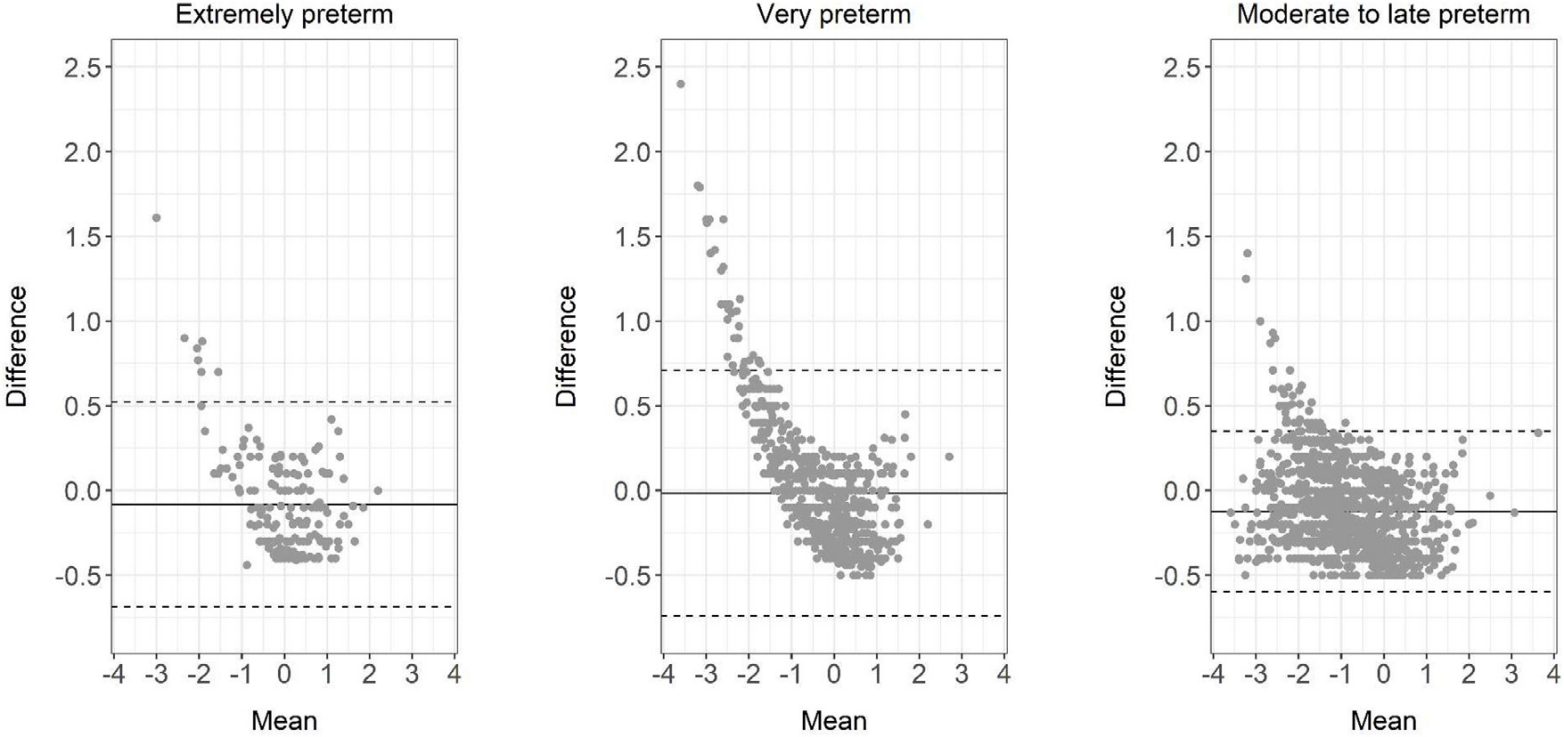
Bland-Altman plots for agreement analysis of birth weight-for-gestational-age Z-scores by prematurity degree between Fenton and INTERGROWTH in preterm infants (2018–2021). CAPTION: In the Bland-Altman plot, the x-axis represents the mean birth weight-for-gestational-age Z-score obtained from the two charts for each infant (Fenton Z-score + INTERGROWTH Z-score) /2), and the y-axis represents the difference between charts Fenton Z -score – INTERGROWTH Z-score. The solid horizontal line indicates the mean bias, and the dashed horizontal lines indicate the limits of agreement. Values above zero indicate higher Z-scores according to Fenton, whereas values below zero indicate higher Z-scores according to INTERGROWTH.

Figure 2 details agreement according to the degree of prematurity. In extremely preterm infants, the mean bias was −0.08 (SD 0.30), with limits of agreement from −0.68 to 0.52; dispersion was reasonably homogeneous along the central range of the X-axis, although more pronounced at the negative extremes (Z-score < −2). In very preterm infants, despite a mean bias closer to zero (−0.02), the SD was the highest among the groups (0.38), yielding wider limits of agreement (−0.74 to 0.71). Among moderate to late preterm infants, the greatest bias was observed (−0.12), yet the SD was the lowest (0.24), with narrower limits of agreement (−0.60 to 0.35), resulting in points concentrated around the mean and few, proportionally, above the upper limit.

Supplemental Figure 2 presents the agreement between charts according to SGA/AGA/LGA status. Since classification may differ between references, subgroups were defined separately for Fenton and INTERGROWTH, resulting in six stratified plots, with detailed agreement estimates provided in Supplemental Table 1.

Within the SGA subgroup, the mean bias was close to zero (0.08 and 0.12 for Fenton and INTERGROWTH, respectively), suggesting overall concordance at the group level. However, this should not be interpreted as indicative of good agreement, given the substantial dispersion observed - reflected by high SD values (0.40 and 0.39), wide limits of agreement, and a considerable number of data points exceeding the upper limit, particularly among neonates with the most negative z-scores. Nevertheless, most SGA infants retained the same classification across charts. The positive mean bias denotes a slight overestimation of Z-scores by Fenton. AGA infants showed the mean bias farthest from zero (−0.14 and −0.16), but lower SDs (0.22 and 0.20), resulting in less dispersion and narrower limits of agreement; only five cases exceeded the upper limit, represented by gray inverted triangles (AGA by Fenton and SGA by INTERGROWTH). The LGA group showed the mean bias closest to zero for Fenton (−0.02), with equally low SD (0.22 for both charts) and limits of agreement containing all cases, confirming the greatest agreement among the three subgroups.

**Figure 3:**
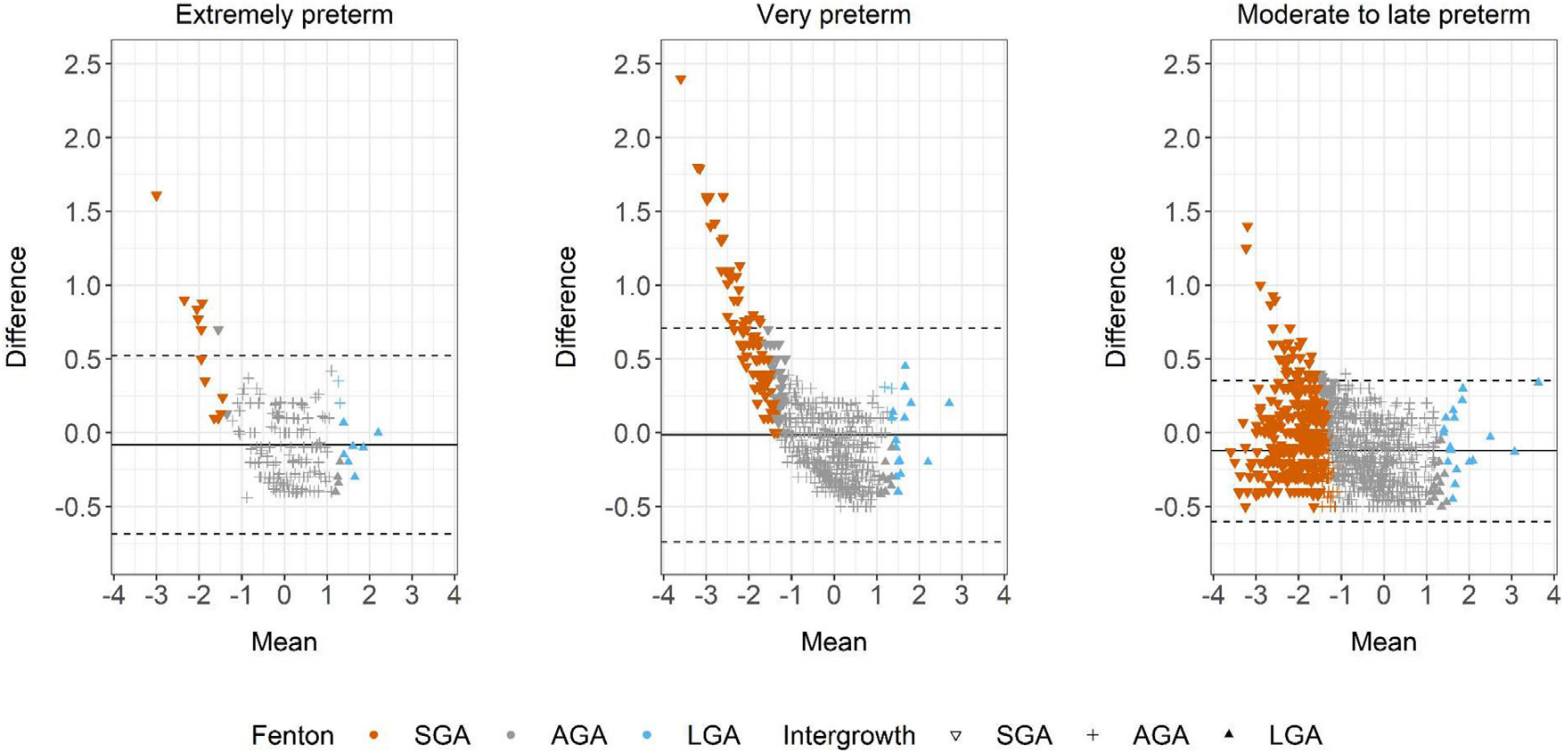
Bland-Altman plots for agreement analysis of birth weight-for-gestational-age Z-scores by prematurity degree and classification as small, appropriate, and large for gestational age between Fenton and INTERGROWTH in preterm infants (2018–2021). CAPTION: In the Bland-Altman plot, the x-axis represents the mean birth weight-for-gestational-age Z-score obtained from the two charts for each infant (Fenton Z-score + INTERGROWTHZ-score) /2), and the y-axis represents the difference between charts Fenton Z-score – INTERGROWTH Z-score. The solid horizontal line indicates the mean bias, and the dashed horizontal lines indicate the limits of agreement. Values above zero indicate higher Z-scores according to Fenton, whereas values below zero indicate higher Z-scores according to INTERGROWTH. Colors indicate classification according to the Fenton chart, whereas symbols indicate classification according to the INTERGROWTH chart. SGA, small for gestational age; AGA, appropriate for gestational age; LGA, large for gestational age.

Figure 3 explores agreement according to degree of prematurity and SGA/AGA/LGA status simultaneously. The main discrepancies occurred in the SGA group, especially among extremely preterm infants - 50% exceeded the upper limit of agreement (6 cases by Fenton and 7 by INTERGROWTH). Among very preterm infants, 32.4% of SGA infants by Fenton (33 cases) and 22.7% by INTERGROWTH (33 cases) also exceeded this limit. Among moderate or late preterm infants, the percentages were lower: 7.9% by Fenton (41 cases) and 8.7% by INTERGROWTH (45 cases). AGA and LGA infants showed few or no cases outside the limits of agreement.

**Figure 4:**
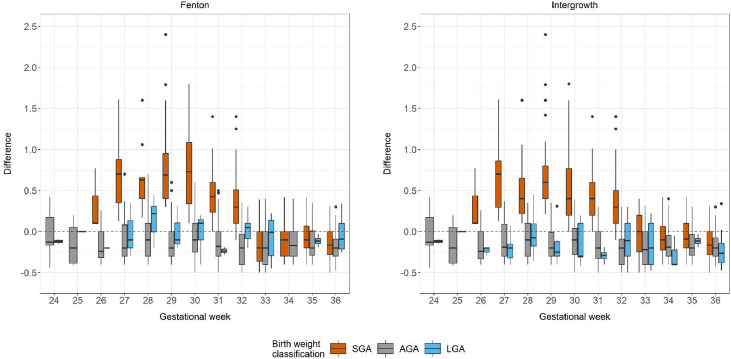
Distribution of differences in birth weight-for-gestational-age Z-scores between Fenton and INTERGROWTH stratified by gestational week and classification as small, appropriate, and large for gestational age in preterm infants (2018–2021). CAPTION: Boxplots show the distribution of differences between birth weight-for-gestational-age Z-scores calculated according to Fenton and INTERGROWTH (Fenton Z−score − INTERGROWTH Z−score) across gestational weeks. Values above zero indicate higher Z-scores according to Fenton, whereas values below zero indicate higher Z-scores according to INTERGROWTH. Boxes represent the interquartile range, horizontal lines within boxes represent medians, whiskers represent the range of non-outlying values, and isolated points indicate outliers. SGA, small for gestational age; AGA, appropriate for gestational age; LGA, large for gestational age.

The analysis of GA as a continuous variable using boxplots in Figure 4 complements the previous results, where this variable was presented in categories. The greatest discrepancies occurred among SGA infants in the 26–32-week range, with systematically higher Fenton values. In AGA and LGA infants, differences were close to zero with lower dispersion. Positive outliers were also identified between 27–32 and 34–36 weeks.

## Discussion

The present study provides a comprehensive agreement evaluation between Fenton and INTERGROWTH charts for birth weight-for-gestational-age Z-scores in preterm infants, using Bland-Altman analysis (a continuous, graphical approach that captures agreement throughout the full distribution), including clinically relevant subgroups defined by prematurity degree and SGA/AGA/LGA status. Overall agreement was reasonable, consistent with previous studies using concordance coefficients. However, deeper analysis revealed meaningful variation according to indicator values and the subgroups considered. The greatest discrepancies occurred in SGA newborns regardless of prematurity degree, though most pronounced among extremely preterm infants. In this subpopulation, a positive bias indicated that the Fenton chart tends to assign systematically higher Z-scores. These findings suggest that global agreement metrics may obscure clinically relevant discrepancies, whereas interchangeable use of the two charts appears more acceptable among moderate to late preterm infants, particularly those classified as AGA or LGA.

Previous studies evaluating agreement between Fenton and INTERGROWTH charts have primarily relied on concordance coefficients and categorical classification (SGA/AGA/LGA), both at birth and/or in the postnatal period (extrauterine growth restriction) [[11], [12], [13],^18^]. Such approaches, however, do not perform simultaneous stratification by prematurity degree and birth weight adequacy, nor do they fully capture the continuous dimension of weight-for-gestational-age Z-score differences, and therefore do not support robust conclusions regarding clinical interchangeability. By applying continuous agreement analysis using Bland-Altman plots, combined with stratification by prematurity degree and SGA/AGA/LGA status, the present study allows a more nuanced interpretation, encompassing not only classification concordance but also the quantification and distribution of discrepancies across the Z-score range within categories and their potential clinical impact.

Stratified analysis by degree of prematurity revealed that disagreement between charts extended beyond extremely preterm infants. In the very preterm group, limits of agreement were wide, with the upper limit approaching values that may be clinically unacceptable, suggesting that individual discrepancies in this stratum can be substantial. Among extremely preterm infants, the mean bias was small, but data dispersion was considerable, reflecting low overall agreement despite the apparent proximity of mean values. A different pattern emerged in moderate to late preterm infants: this group showed a greater systematic bias, with INTERGROWTH consistently assigning higher Z-scores than Fenton, but also the lowest SD, indicating more predictable and consistent between-method differences. This distinction matters clinically: systematic bias with low variability is more transparent and manageable than random discrepancy with wide dispersion. These findings suggest that, although interchangeability between charts may be relatively more acceptable in moderate to late preterm infants, caution remains warranted across all prematurity strata, particularly when individual clinical decisions depend on precise Z-score values.

This concern is further amplified when considering SGA infants, a population in which precise Z-score classification carries direct implications for nutritional diagnosis and intervention. In this subgroup, especially those born extremely preterm, dispersion was markedly higher and limits of agreement were wide, indicating that individual Z-score differences frequently reached magnitudes of potential clinical relevance. Differences approaching 0.8SD or more, as observed in this subgroup, correspond to thresholds used in the diagnosis of mild malnutrition according to the consensus published by the Academy of Nutrition and Dietetics [19]. Critically, even when the mean bias was close to zero, substantial variability persisted at the individual level, underscoring that mean differences alone are insufficient to support interchangeability between charts. This distinction is clinically meaningful: nutritional assessment and monitoring in neonatal practice frequently rely on changes in Z-scores over time (delta Z), and variations of this magnitude between charts may lead to misinterpretation of growth trajectories, particularly in more immature SGA infants. Switching between charts during follow-up may artificially amplify or attenuate perceived growth changes, potentially resulting in inappropriate adjustments in nutritional management. Therefore, apparently modest differences between charts may carry meaningful implications for longitudinal nutritional assessment and clinical decision-making when charts are used interchangeably.

Although Fenton and INTERGROWTH are both widely used in clinical practice, their methodological foundations differ substantially: Fenton derives from a meta-analysis of large population-based studies, whereas INTERGROWTH reflects growth under optimal conditions [20]. These differences help explain the discordant classifications observed in this study, particularly at the extremes of gestational age. Notably, Fenton tended to assign higher Z-scores than INTERGROWTH in the SGA subgroup, especially between 26 and 32 weeks. This pattern may reduce SGA birth rate, potentially delaying the recognition of higher-risk infants and impairing earlier nutritional intervention and monitoring. The consequences of such divergence are not merely statistical: in clinical settings with a high prevalence of extremely preterm or SGA infants, such as Brazilian public NICUs, the choice of growth reference may directly impact clinical management [6,7]. Health care providers should be aware that Z-score values are not directly interchangeable across charts and should interpret them within the context of the chosen reference, ideally maintaining a single, consistent chart throughout hospitalization and follow-up. These findings highlight that agreement between growth charts should not be interpreted solely based on categorical concordance, but rather on the potential clinical consequences of continuous differences, especially in high-risk neonatal populations.

This study has several limitations that should be considered when interpreting the findings. As a retrospective analysis based on secondary data, it was not possible to control for all clinical and nutritional variables that may influence growth. Gestational age estimation relied on multiple sources, including clinical methods such as Capurro and Ballard in some cases, which may introduce measurement variability, particularly among the most immature infants. Furthermore, the exclusion of infants hospitalized for fewer than 14 days, those admitted exclusively to Kangaroo Intermediate Care Units, and those who died during hospitalization may have introduced selection bias, limiting generalizability to lower-risk or more severe populations. Anthropometric measurements were obtained by different professionals, which may also affect data consistency.

Despite these limitations, the study has important strengths. It includes a large sample with representation across all degrees of prematurity and applies a robust analytical framework that goes beyond traditional categorical comparisons. The use of Bland-Altman analysis combined with clinically meaningful stratifications enabled the identification of agreement patterns that would likely remain undetected using conventional methods. This approach provides a more nuanced understanding of how growth charts perform in real-world clinical populations.

Graphical agreement analysis between Fenton and INTERGROWTH charts demonstrated that, although reasonable overall correspondence exists, clinically relevant differences emerge when stratifying by prematurity degree and growth classification. Disagreement extends beyond extremely preterm infants, affecting all prematurity strata with distinct patterns: greater dispersion and less predictable differences among extremely and very preterm infants, and more consistent but systematically biased differences among moderate to late preterm infants.

The most pronounced inconsistencies were observed in SGA infants, particularly among the most immature, in whom differences frequently reached magnitudes of potential clinical relevance. In this subgroup, the tendency of the Fenton chart to assign higher Z-scores may attenuate the apparent severity of birth weight deficit and influence nutritional assessment and management. Notably, even in the presence of minimal mean bias, wide limits of agreement indicate that the charts are not interchangeable at the individual level.

Therefore, growth reference selection should be guided not only by normative considerations but also by the clinical context and intended use, with consistent application of a single chart being essential for longitudinal assessment. This is particularly relevant in high-complexity neonatal settings such as Brazilian public NICUs, especially for more immature SGA infants.

The analytical approach adopted allowed identification of variability patterns not captured by traditional categorical methods, reinforcing the relevance of continuous agreement analysis in neonatal growth evaluation. Further studies should investigate the longitudinal impact of these discrepancies on growth trajectories to better inform evidence-based practice.

## Data availability statement

The data that support the findings of this study are available from the corresponding author.

## Financial support

Self-funded.

## Conflicts of interest

The authors declare no conflicts of interest.

## Appendix Supplementary materials

Image, application 1

## Footnotes

Supplementary material associated with this article can be found in the online version at doi:10.1016/j.jped.2026.101575.
