# Supplementary material for: Agreement between Fenton and intergrowth curves in assessing birth weight of preterm infants: Bland-Altman analysis by degree of prematurity and birth weight for gestational age
Source: J Pediatr (Rio J). 2026 Jun 26;102(5):101575. doi: 10.1016/j.jped.2026.101575 (PMC13321015; doi:10.1016/j.jped.2026.101575)
Supplement: Supplementary file 1 [file mmc1.docx]

**JPED-D-25-00559_Supplementary Material**

**Supplemental Table 1** Mean bias, standard deviation, and limits of agreement from Bland-Altman analyses comparing Fenton and INTERGROWTH birth weight-for-gestational-age Z-scores, overall, by prematurity degree, and by birth weight adequacy subgroups defined separately according to each growth chart (2018–2021).

| **Subgroup** | Mean bias | SD | -2SD | +2SD |
| --- | --- | --- | --- | --- |
| Overall | -0.08 | 0.29 | -0.67 | 0.50 |
| **Prematurity degree** |  |  |  |  |
| Extremely preterm | -0.08 | 0.30 | -0.68 | 0.52 |
| Very preterm | -0.02 | 0.36 | -0.74 | 0.71 |
| Moderate to late preterm | -0.12 | 0.24 | -0.6 | 0.35 |
| **Birth weight adequacy** | **Fenton** | | | |
| SGA | 0.08 | 0.40 | -0.73 | 0.89 |
| AGA | -0.14 | 0.22 | -0.58 | 0.29 |
| LGA | -0.02 | 0.22 | -0.46 | 0.42 |
| **Birth weight adequacy** | **INTERGROWTH** | | | |
| SGA | 0.12 | 0.39 | -0.65 | 0.90 |
| AGA | -0.16 | 0.20 | -0.56 | 0.24 |
| LGA | -0.16 | 0.22 | -0.61 | 0.28 |

SGA, small for gestational age; AGA, appropriate for gestational age; LGA, large for gestational age.

**Supplemental Figure 1** Bland-Altman plot for overall agreement analysis of birth weight-for-gestational-age Z-scores between Fenton and INTERGROWTH in preterm infants (2018-2021).


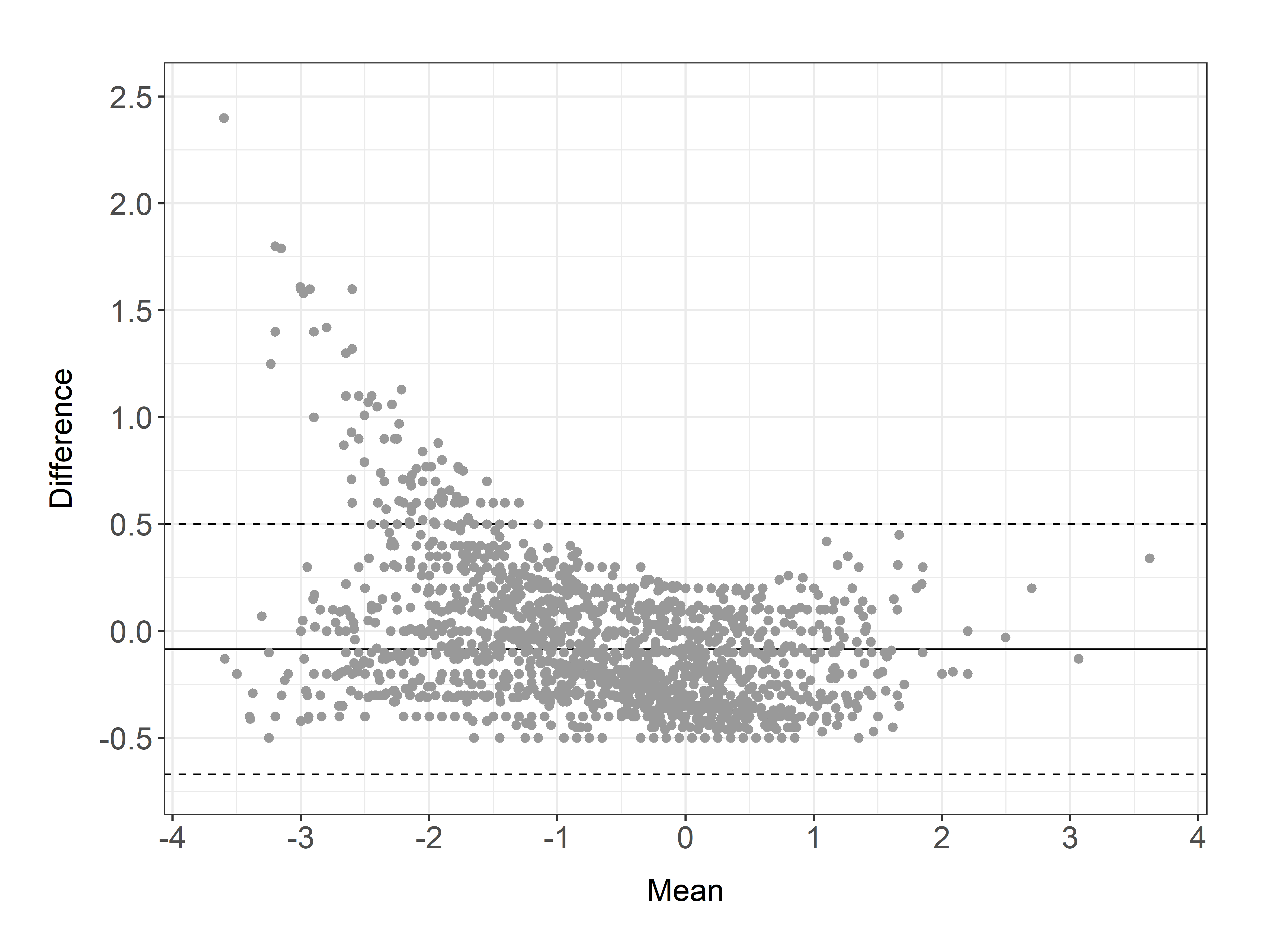


CAPTION: In the Bland-Altman plot, the x-axis represents the mean birth weight-for-gestational-age Z-score obtained from the two charts for each infant (Fenton Z-score + INTERGROWTH Z-score) /2), and the y-axis represents the difference between charts Fenton Z-score - INTERGROWTH Z-score. The solid horizontal line indicates the mean bias, and the dashed horizontal lines indicate the limits of agreement.

**Supplemental Figure 2** Bland-Altman plot for agreement analysis of birth weight-for-gestational-age Z-scores by classification as small, appropriate, and large for gestational age between Fenton and INTERGROWTH in preterm infants (2018–2021).


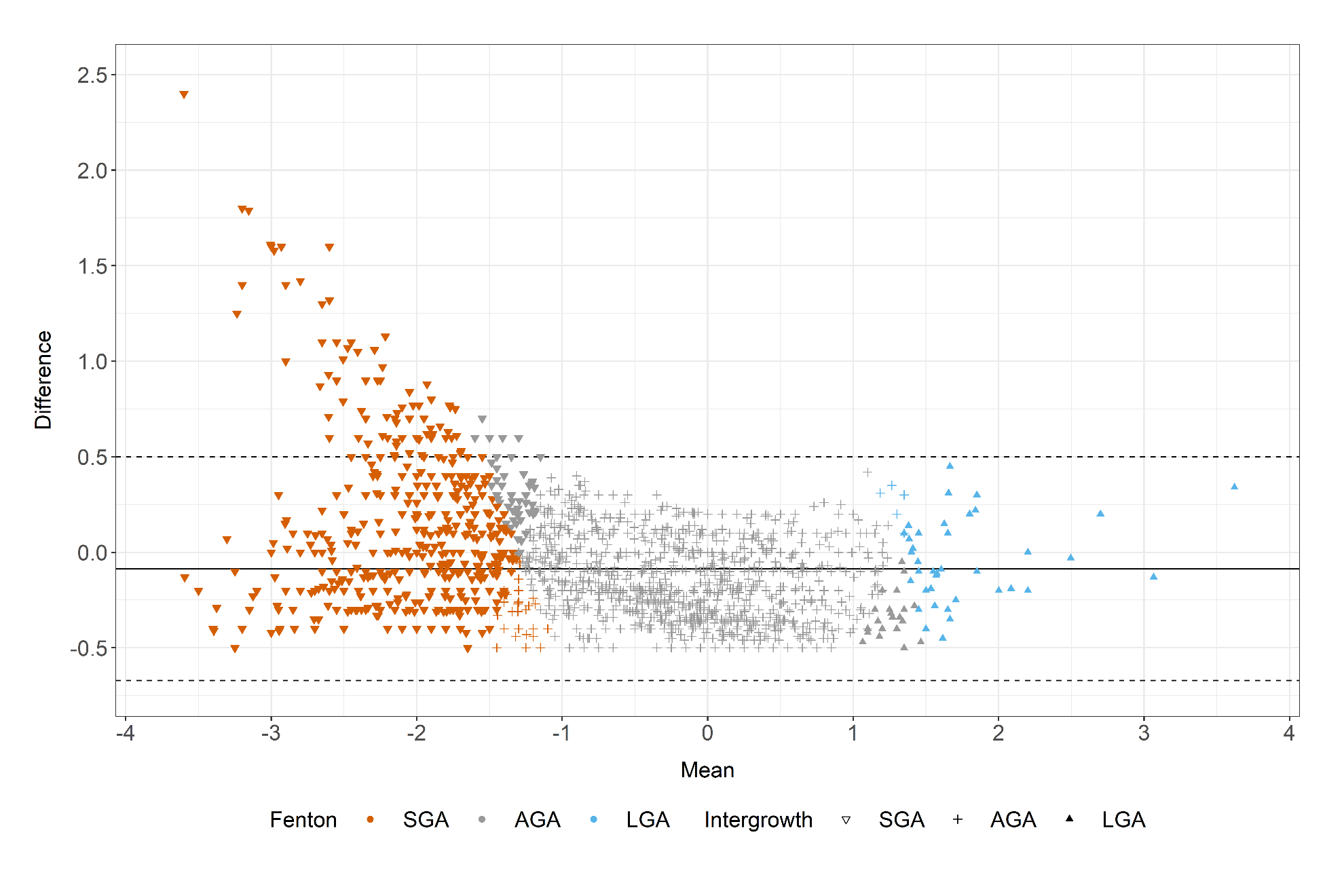


CAPTION: In the Bland-Altman plot, the x-axis represents the mean birth weight-for-gestational-age Z-score obtained from the two charts for each infant (Fenton Z-score + INTERGROWTH Z-score) /2), and the y-axis represents the difference between charts Fenton Z-score - INTERGROWTH Z-score. The solid horizontal line indicates the mean bias, and the dashed horizontal lines indicate the limits of agreement.

Values above zero indicate higher Z-scores according to Fenton, whereas values below zero indicate higher Z-scores according to INTERGROWTH. Colors indicate classification according to the Fenton chart, whereas symbols indicate classification according to the INTERGROWTH chart. SGA, small for gestational age; AGA, appropriate for gestational age; LGA, large for gestational age.
